# Co (II) Boron Imidazolate Framework with Rigid Auxiliary Linkers for Stable Electrocatalytic Oxygen Evolution Reaction

**DOI:** 10.1002/advs.201801920

**Published:** 2019-03-18

**Authors:** Tian Wen, Yao Zheng, Jian Zhang, Kenneth Davey, Shi‐Zhang Qiao

**Affiliations:** ^1^ State Key Laboratory of Structural Chemistry Fujian Institute of Research on the Structure of Matter Chinese Academy of Sciences Fuzhou Fujian 350002 P. R. China; ^2^ School of Chemical Engineering The University of Adelaide Adelaide SA 5005 Australia

**Keywords:** boron imidazolate frameworks, confinement effects, metal–organic frameworks, oxygen evolution reaction, zeolite

## Abstract

Metal–organic frameworks (MOFs) have significant potential for practical application in catalysis. However, many MOFs are shown to be sensitive to aqueous solution. This severely limits application of MOFs in electrocatalytic operations for energy production and storage. Here, a Co (II) boron imidazolate framework CoB(im)_4_(ndc)_0.5_ (**BIF‐91**, im = imidazolate, ndc = 2,6‐naphthalenedicarboxylate) that is rationally designed and successfully tested for electrocatalytic application in strong alkaline (pH ≈ 14) solution is reported. In such a BIF system, the inherent carboxylate species segment large channel spaces into multiple domains in which each single channel is filled with ndc ligands through the effect of zeolite channel confinement. These ligands, with strong C—H···π interaction, act as a rigid auxiliary linker to significantly enhance the structural stability of the **BIF‐91** framework. Additionally, the π‐conjugated effect in **BIF‐91** stabilizes dopant Fe (III) at the atomic scale to construct Fe‐immobilized **BIF‐91** (**Fe@BIF‐91**). Due to the synergistic effect between Fe (III) guest and Co (II) in the framework, the **Fe@BIF‐91** acts as an active and stable electrocatalyst for the oxygen evolution reaction in alkaline solution.

The features unique to metal–organic frameworks (MOFs), of large surface area and tailorable chemical functionalities, make them attractive for applications in gas separation and catalysis.^1^, ^2^, ^3^, ^4^, ^5^, ^6^, ^7^, ^8^ However, a loss of these functionalities in aqueous electrolytes (strong acid or alkali) severely limits application of MOFs in energy‐related electrocatalysis. The stability of MOFs is attributed to their inherent frameworks, namely, coordinated metal ions, hydrophobicity of organic ligands, and metal–ligand coordination geometry.^9^, ^10^ Several strategies however have been developed to prepare stable MOFs in aqueous solution. For example, postsynthetic metal metathesis (PSM) has been extensively applied to d^10^ metals‐based MOFs.^11^ In addition, stable paddle‐wheel type MOFs can be made through modular and stepwise synthesis.^12^ However, these two methods are complicated, and involve a period of several weeks. As a consequence, only a limited number of MOFs have been reported which are used as electrocatalysts in acid and alkaline solution.^13^, ^14^, ^15^ This is because transition‐metal‐based MOFs readily collapse in these relative harsh environments. The result is a destroyed host‐framework and loss of active sites. Therefore, designing new MOFs with intrinsic stability is important for their applications in electrocatalysis field.

Boron imidazolate frameworks (BIFs) are a subclass of MOFs that are made from boron imidazolate ligands and d^10^ metal cations.^16^ Uniformly distributed 3/4‐connected boron components keep the framework rigid. Some specially designed BIFs, for example, **BIF‐89**, are therefore stable in common solvents such as water and dimethylformamide (DMF). This means these are, potentially, alkaline‐resistant electrocatalysts for oxygen evolution reaction (OER).^17^ **BIF‐89** however showed poor OER activity because the coordinated Cd atoms in the framework are electrocatalytically inert. Fe^3+^ immersed **BIF‐89** (**Fe@BIF‐89**) works as an effective OER electrocatalyst in alkaline solution because advantage is made of the uncoordinated carboxylic groups to stabilize the incorporation of the Fe^3+^ cation. It is widely known that Co‐ or Fe‐based materials exhibit good electrocatalytic performance because of highly reactive catalytic sites.^18^, ^19^, ^20^, ^21^ We proposed therefore to rationally design a bimetallic system containing Co‐based BIFs incorporated with Fe^3+^ to achieve synergistically enhanced OER activity.

We report a new Co‐based red‐block crystal of CoB(im)_4_(ndc)_0.5_ (**BIF‐91**, im = imidazolate, ndc = 2, 6‐naphthalenedicarboxylate) that was successfully synthesized by reaction of H_4_ndc with cobalt (II) acetate tetrahydrate and presynthesized KB (im)_4_ ligand. Notably, the presence of bridging ndc acid ligands in the **BIF‐91** molecular channels decreased its porosity and increased the density of coordination bonds. **BIF‐91** therefore kept its original crystallinity in strong alkaline solution. The rich five/six‐membered rings with π‐conjugated effects in the **BIF‐91** framework ensure a favorable atomic level absorption of Fe^3+^ cations in its channels. Because of a bimetallic synergistic effect of inherent Co^2+^ and guest Fe^3+^ cations, **BIF‐91 (Fe@BIF‐91)** exhibited enhanced electrochemical activity when compared with Fe‐containing only **Fe@BIF‐89**. This is also true when compared with benchmark metal catalysts for water oxidation under strong alkaline conditions.

The **BIF‐91** framework was prepared by self‐assembly of cobalt salts, boron imidazolate ligands, and rigid auxiliary linkers via solvothermal method at 80 °C for 4 d (Figure S1, Supporting Information). Single‐crystal X‐ray diffraction analysis showed that **BIF‐91** crystallized in *P21/c* space group in which each Co^2+^ center adopts a CoN_4_O_2_ distorted octahedral coordination geometry and is bonded by four (4) nitrogen atoms in B(im)_4_^−^ ligands and two O atoms in ndc^2−^ linker (**Figure** **1**a, and Table S1, Supporting Information). In the **BIF‐91** framework, the six‐coordinated and five‐connected center is linked with ndc^2−^ and B(im)_4_^−^ ligands into a 3D porous framework (Figure 1b). Importantly, as demonstrated in Figure 1c, there are obvious C—H···π interactions between the ndc^2−^ linker and B(im)_4_^−^ ligands in the channel. These act to strengthen the stability of whole framework. Thermogravimetric analysis (TGA) showed only a slight weight change prior to 300 °C (due to the solvent loss), indicating the dense framework was of good thermal stability (Figure S2, Supporting Information). Interestingly, the **BIF‐91** framework can be considered as an eight‐membered ring molecular sieve (≈9.9 × 14.4 Å) when the bridging ndc acid ligands serve as redundant bonds (Figure 1d, and Figures S3 and S4, Supporting Information).

**Figure 1:**
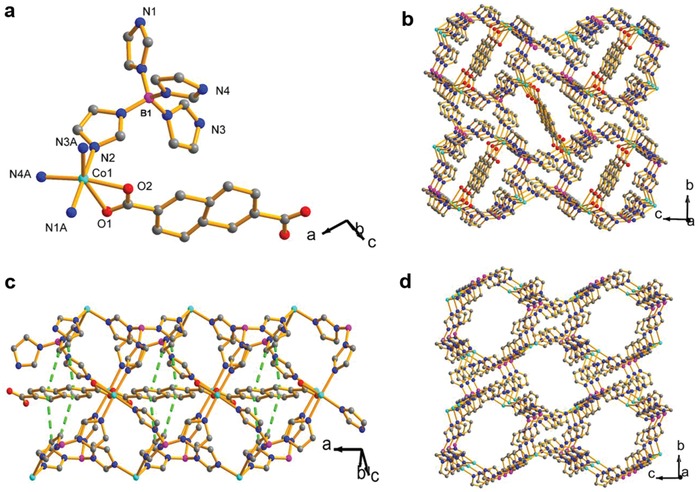
a) Coordination environment of Co (II) in **BIF‐91** framework. b) Schematic of the 3D framework of **BIF‐91**. c) Stabilization effect of ndc acidic ligands by C—H···π interactions indicated by dashed green bonds (length ≈ 3.8 Å). d) Simplified zeolitic framework of **BIF‐91** without rigid auxiliary linker. Color code: B in purple, Co in pale blue, N in blue, O in red, and C in black.

Generally, the instability of MOFs in aqueous solution results because the metal node is not sufficiently stable to support an open coordination framework. Specifically, these coordinated metal cations with isolated, single‐pyramidal coordination geometries, have relatively high reactivity with adsorption affinities and catalytic properties. To address this, a ndc^2−^ organic linker was introduced to fill the 1D channel in **BIF‐91** framework. Because the π‐electron‐rich 2,6‐naphthalenedicarboxylic acid serves as a rigid linker, it decreases the porosity and increases stability of the **BIF‐91** framework. To verify the chemical stability of **BIF‐91** compared with other typical zeolite‐type MOFs, for example, **BIF‐22** and **ZIF‐67** with large cavities, the powder X‐ray diffraction (PXRD) patterns of fresh samples, and samples following immersion in 1 m KOH aqueous solution were compared.^22^, ^23^ As is seen in **Figure** **2**, the characterizes of PXRD are unchanged with **BIF‐91**, but have all but disappeared with the two selected example MOFs. This phenomena can be explained at the molecular level because the large porous channel framework of **BIF‐91** is partitioned into two domains via bridging dicarboxylate ndc ligands. The result is therefore a high stability in strong alkaline solution.

**Figure 2:**
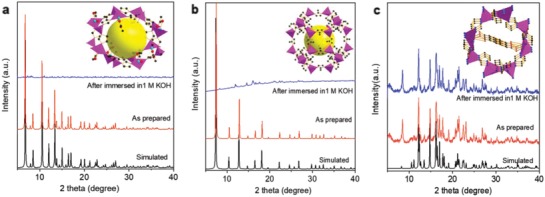
a–c) PXRD patterns of **BIF‐22**, **ZIF‐67**, and **BIF‐91** MOFs before and after immersion in 1 m KOH solution. Insets show the polyhedral model of single‐cavity frameworks.

As is shown in Figure 1b, **BIF‐91** with π conjugation in the channel adsorbs positively charged cations from solution. The PXRD pattern confirms therefore that the porous framework of **BIF‐91** was unchanged after capturing 4.3% Fe^3+^ cations from the FeCl_3_ solution (**Figure** **3**a). X‐ray photoelectron spectroscopy (XPS) survey spectrum confirmed the coexistence of Fe and Co atoms in the **BIF‐91** framework (Figure 3b). Specifically, the high‐resolution XPS spectra of Co 2p confirmed Co^2+^ species in **Fe@BIF‐91** (two main peaks at, respectively, 873.9 and 856.3 eV). This strongly suggests the chemical state of Co was unchanged after deposition of Fe^3+^ (Figure 3c). The Fe 2p region spectrum suggests the chemical state of the Fe in **Fe@BIF‐91** is Fe^3+^, with two main peaks at, respectively, 712.7 and 725.9 eV (Figure 3d). Further, the transmission electron microscope (TEM) element mapping showed well‐distributed Fe species in the **BIF‐91** framework (Figure 3e–g). The morphology of **Fe@BIF‐91** after immobilization is smooth, with no lattice fringe of crystallized Fe or, Co oxides, detected in the high‐resolution TEM image. This finding indicates that there was no aggregation or clustering of the bulk metal nanoparticles (Figure 3h).

**Figure 3:**
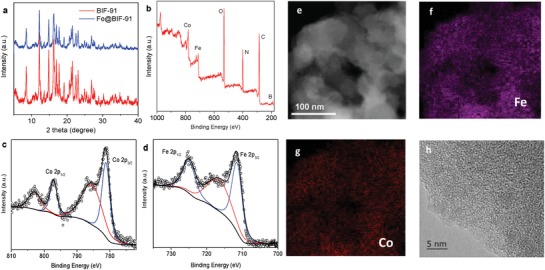
a) PXRD patterns of **BIF‐91** and **Fe@BIF‐91** samples. b–d) XPS survey, Co 2p and Fe 2p high‐resolution spectra of **Fe@BIF‐91**. e–g) TEM imaging and corresponding EDX mapping of Fe and Co in **Fe@BIF‐91**. h) High‐resolution TEM image of **Fe@BIF‐91**.

Linear sweep voltammetry (LSV) was performed in strong alkaline solution (pH ≈ 14) to quantitatively investigate the electrocatalytic activity of **Fe@BIF‐91** for OER. The **Fe@BIF‐91** electrocatalyst displayed an overpotential of 350 mV at 10 mA cm^−2^ and a Tafel slope of 71 mV dec^−1^, which are significantly less than that of ordinary **BIF‐91** (**Figure** **4**a, and Figure S5, Supporting Information). The electrochemical impedance spectrum (EIS) analysis, and electrochemical double‐layer capacitance (*C*_dl_) test, further confirmed that the function of Fe^3+^ in enhancing OER activity is through decreasing the charge transfer impedance and enlarging the catalytically active surface area in comparison with **BIF‐91** (Figures S6 and S7, Supporting Information). It should be noted that this excellent OER performance is meaningfully greater than (benchmark) RuO_2_ and is similar to that of recently reported MOFs catalysts (Figure 4a, and Table S2, Supporting Information).^12^, ^17^, ^24^, ^25^, ^26^, ^27^, ^28^, ^29^, ^30^ Importantly, the Fe, Co co‐contained **Fe@BIF‐91** electrocatalyst, showed enhanced OER activity greater than Fe‐containing‐only **Fe@BIF‐89**. This indicates a synergistic catalysis between Fe and Co cations in the newly developed **Fe@BIF‐91** framework. This effect is more significant with increasing Fe^3+^ immersed concentration in the **BIF‐91** (Figure 4b).

**Figure 4:**
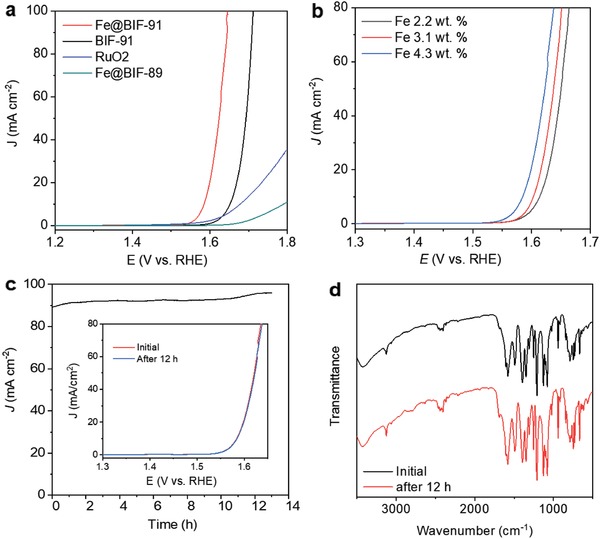
a) OER polarization curves of various BIF‐based electrocatalysts and bench marker in 1 m KOH aqueous solution. b) OER polarization curves for a series of **Fe@BIF‐91** electrocatalysts with varying Fe^3+^ concentration. c) Chronoamperometric curves for **Fe@BIF‐91** electrocatalyst in 1 m KOH aqueous solution. Inset: LSV plots of **Fe@BIF‐91** electrocatalyst before and after a 12 h stability test. d) FTIR patterns of **Fe@BIF‐91** electrocatalyst before and after OER stability test.

The chronoamperometry, scanning electron microscopy (SEM, Figure S8, Supporting Information), and LSV measurements showed that **Fe@BIF‐91** catalysts produce a stable anodic current under after 12 h of OER (Figure 4c). This finding clearly demonstrates its electrochemical stability under OER. The Fourier‐transform infrared (FTIR) spectroscopies of **Fe@BIF‐89** before and after reaction were compared to evaluate structural alkaline resistance. As is shown in Figure 4d, the absorption peak at ≈1576 cm^−1^, attributed to the stretching vibration of C=N in the imidazole ring, and the stretching peaks of carboxylate group and arcmatic ring at 1288–1700 cm^−1^, are all preserved. This demonstrates that the **BIF‐91** framework was unchanged after OER.

In conclusion, new alkaline‐stable neutral zeolite‐type Co‐based BIFs were rationally designed and successfully synthesized for OER application. A C—H···π interaction between partial imidazolate and 2,6‐naphthalenedicarboxylic ligands was formed because of the well‐designed confinement effect in each porous channel. As a result, the newly developed **BIF‐91** framework exhibited excellent alkaline resistance when compared with other BIFs‐type materials such as **BIF‐22** and **ZIF‐67**. Due to a synergistic catalysis between immersed Fe^3+^ and inherent Co^2+^ in the framework, the **Fe@BIF‐91** electrocatalyst demonstrated significantly enhanced OER activity compared with ordinary **BIF‐89** and Fe‐containing‐only **Fe@BIF‐89** and existing MOF materials in strong alkaline solution (pH ≈ 14). The confinement effect and π‐conjugated framework of **BIF‐91** has potential application for adsorbing active Fe^3+^ ions at atomic level. This creates practical potential for new designs of stable MOFs to capture active species. These findings will be of immediate benefit to guide rational design of active bimetal MOFs for application in electrocatalysis.

## Conflict of Interest

The authors declare no conflict of interest.

## Supporting information

Supplementary
