# Supplementary material for: Co (II) Boron Imidazolate Framework with Rigid Auxiliary Linkers for Stable Electrocatalytic Oxygen Evolution Reaction
Source: Adv Sci (Weinh). 2019 Mar 18;6(9):1801920. doi: 10.1002/advs.201801920 (PMC6498129; doi:10.1002/advs.201801920)
Supplement: Supplementary file 1 — Supplementary [file ADVS-6-1801920-s001.pdf]

Copyright WILEY-VCH Verlag GmbH & Co. KGaA, 69469 Weinheim, Germany, 2019.

Supporting Information

for *Adv. Sci.,* DOI: 10.1002/advs.201801920

Co (II) Boron Imidazolate Framework with Rigid Auxiliary Linkers for Stable Electrocatalytic Oxygen Evolution Reaction

*Tian Wen, Yao Zheng, Jian Zhang, Kenneth Davey, and Shi-Zhang Qiao**

Supporting Information

Co (II) Boron Imidazolate Framework with Rigid Auxiliary Linkers for Stable Electrocatalytic Oxygen Evolution Reaction

Tian Wen,[a b] Yao Zheng[b] Jian Zhang,[a] Kenneth Davey[b] and Shi-Zhang Qiao*[b]

[a] T. Wen, Prof. J. Zhang

State Key Laboratory of Structural Chemistry, Fujian Institute of Research on the Structure of Matter, Chinese Academy of Sciences, Fuzhou, Fujian 350002, P. R. China.

[b] T. Wen, Dr. Y. Zheng, Dr. K. R. Davey, Prof. S. Z. Qiao

School of Chemical Engineering, The University of Adelaide Adelaide, SA 5005, Australia.

E-mail: s.qiao@adelaide.edu.au

1. Single-crystal structural determination

Single crystals of **BIF-91** were selected and glued to a thin, glass-fibre. Crystallographic data of **BIF-91** were collected on a Mercury, single crystal diffractometer equipped with graphite-monochromatic Mo Kα radiation (λ = 0.71073 Å) at room-temperature. The structures were solved by direct methods and refined by full-matrix least-squares on F2. Crystal data as well as the details of data collection and refinement for **BIF-91** are summarized as Table S1. All powder X-ray diffraction (PXRD) analyses were performed on a Rigaku Dmax2500 diffractometer with Cu Kα radiation (λ = 1.54056 Å) using a step size of 0.05°. Thermogravimetric analyses (TGA) were performed on a Mettler Toledo TGA/SDTA 851e analyzer using a heating rate of 10 °C min^{-1} under N_2 atmosphere. Elemental analyses (EA) for C, H, and N were done on an EA1110 CHNS0 CE elemental analyzer. Fourier transform infrared (FT-IR) spectra were taken on Nicolet Magna 750 FT−IR spectrometer in the 4000 to 500 cm^{-1} region.

2. Electrochemical characterization

OER measurements were performed in a three-electrode glass cell. The data were recorded using a CHI 760D bipotentiostat (CH Instruments, Inc., USA). The synthesized samples were used as the working electrode. The reference electrode was Ag/AgCl in 4 M KCl solution and the counter electrode was a platinum wire. The current density was normalized to the geometrical surface area and the measured potential vs. Ag/AgCl was converted to a reversible hydrogen electrode (RHE) scale. A flow of N_2 was maintained over the electrolyte (1.0 M KOH) during electrochemical measurements. The polarization curves were recorded with a scan rate of 5 mV s^{-1}. The working electrodes were scanned several times before data for polarization curves were collected. Electrochemical impedance spectroscopy (EIS) were measured by applying an AC voltage in the frequency range of 100000 to 1 Hz.

3. Synthesis of BIF-91

2,6-naphthalenedicarboxylate (0.0266 g), Cobalt (II) acetate tetrahydrate (0.0278 g), $KB(im)_4$ (0.0217 g), in a N, N-dimethylformamide (4 mL) / 2-amino-1-butanol/ (1.5 mL) / CH_3CN (1.5 mL) solution were placed in a 20 mL vial. The sample was heated at 80 °C for four days, and cooled to room temperature. Following washing with ethanol and distilled water, red block crystals were obtained (11 % yield).

4. Synthesis of Fe@BIF-91

The as-synthesized **BIF-91** samples were washed with distilled water and methanol before

immersion in $FeCl_3$ (15 mM) for 24, 5, and 3 h, respectively. Further, ICP measurement demonstrated Fe contents were, respectively, 4.3, 3.1 and 2.2 %.

5. Synthesis of BIF-22

A mixture of Cobalt (II) acetate tetrahydrate (0.120 g), $KB(im)_4$ (0.160g), and N,N-dimethylformamide (3 mL) was sealed in a 20 mL vial and heated to 120 °C for 1 day, and cooled to room temperature.

6. Synthesis of ZIF-67

In a typical synthesis, 2-methylimidazole (4 mmol) and $Co(NO_3)_2{\cdot}6H_2O$ (1 mM) was dissolved in 8 mL N,N-dimethylformamide, then placed in a 23 mL Teflon-lined stainless-steel autoclave, which was heated at 120 °C for 72 h. A purple product was obtained.

7. The catalyst coated on glassy carbon electrode

Typically, the as-synthesized **Fe@BIF-91** was ultrasonically dispersed in the mixture of 1 mL of Nafion solution (0.05 wt.% water solution) and then transferred onto the glassy carbon electrode with a loading of ~ 0.23 mg cm^{-2}. The resulting electrode (**Fe@BIF-91@GC**) was dried in air for 5 h. The preparation of other catalysts on the glassy carbon electrode was the same as that for **Fe@BIF-91@GC**.

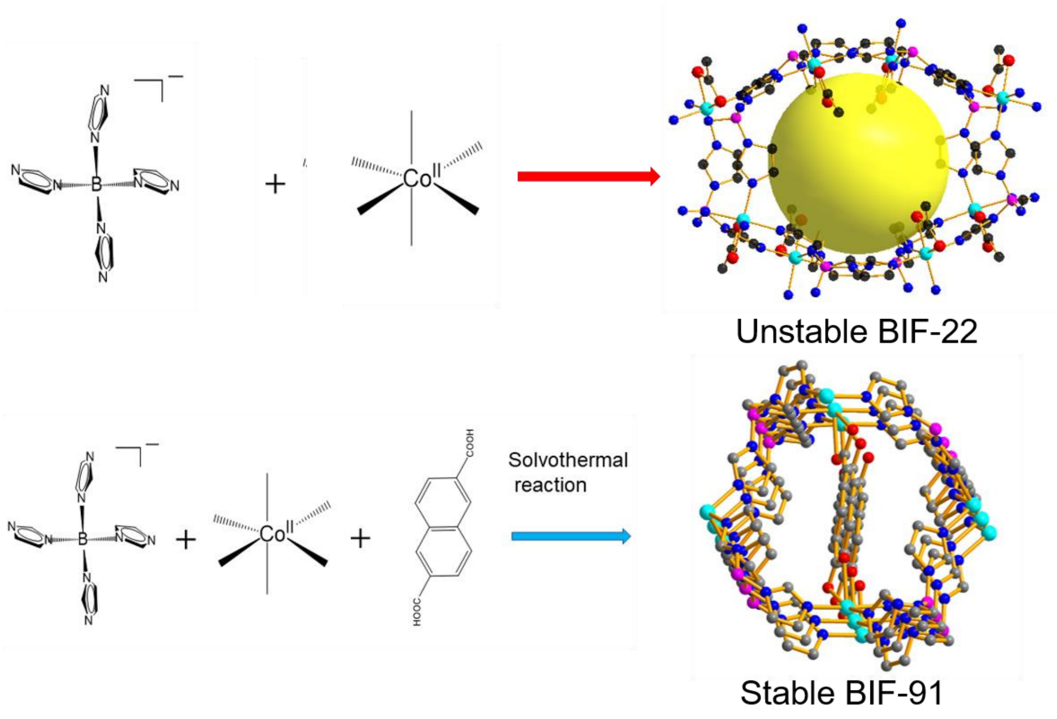

Figure S1. Synthesis of **BIF-91** and **BIF-2**2 via a dissolution-crystallization mechanism under solvothermal conditions. Color code: B in purple, Co in pale blue, N in blue, C in grey (**BIF-91**) and black (**BIF-22**).

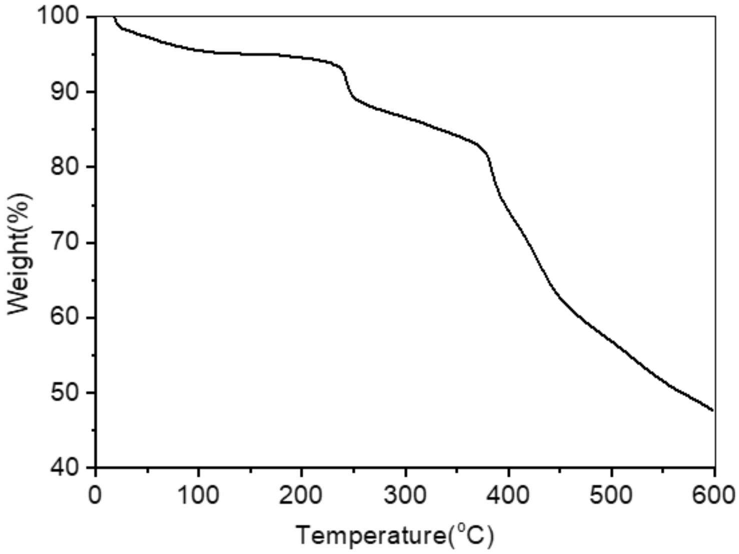

Figure S2. Thermogravimetric profiles of **BIF-91** under N_2.

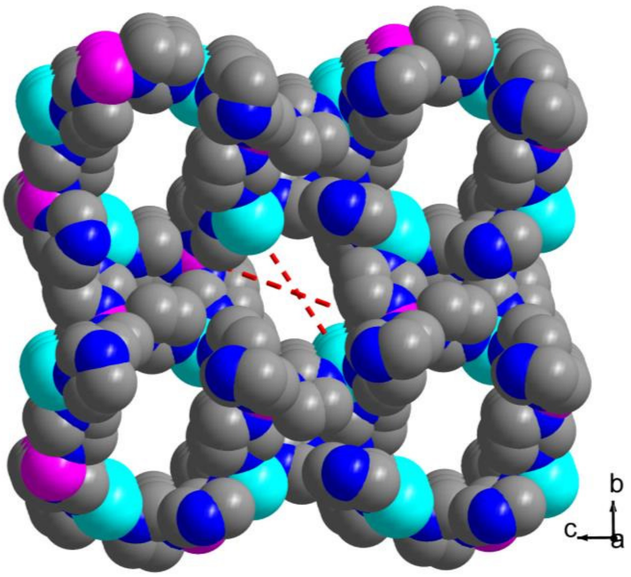

Figure S3. View of molecular sieve structures (simplified eight-membered ring, ~9.9 x14.4 Å). Color code: B in purple, Co in pale blue, N in blue and C in grey.

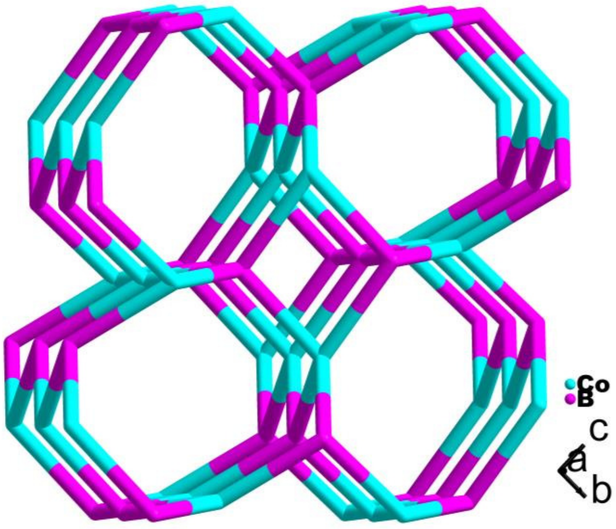

Figure S4. View of typical zeolite BCT topology of simplified **BIF-91**.

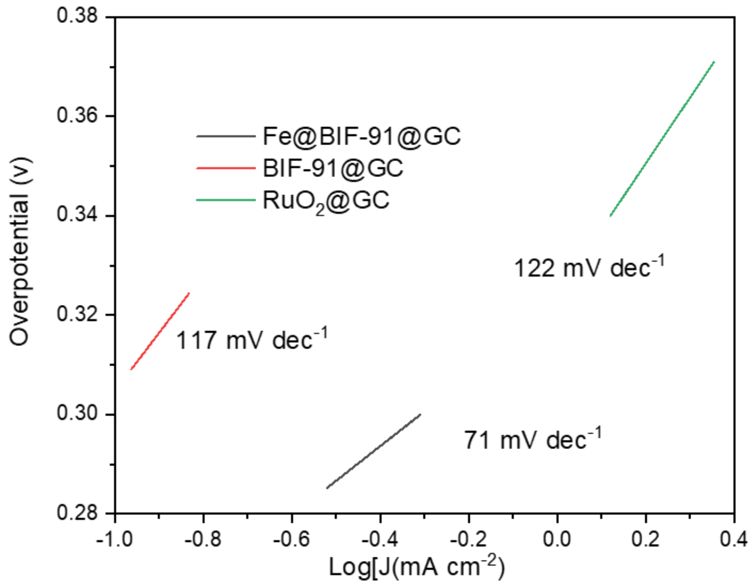

Figure S5. Tafel plots of catalysts in 1M KOH aqueous solution.

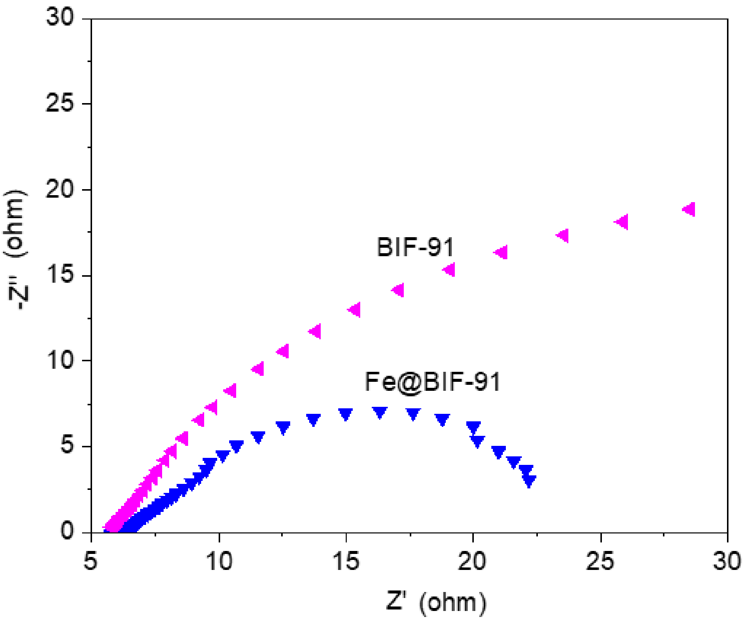

Figure S6. EIS of **BIF-91** and **Fe@BIF-91** electrocatalysts under OER.

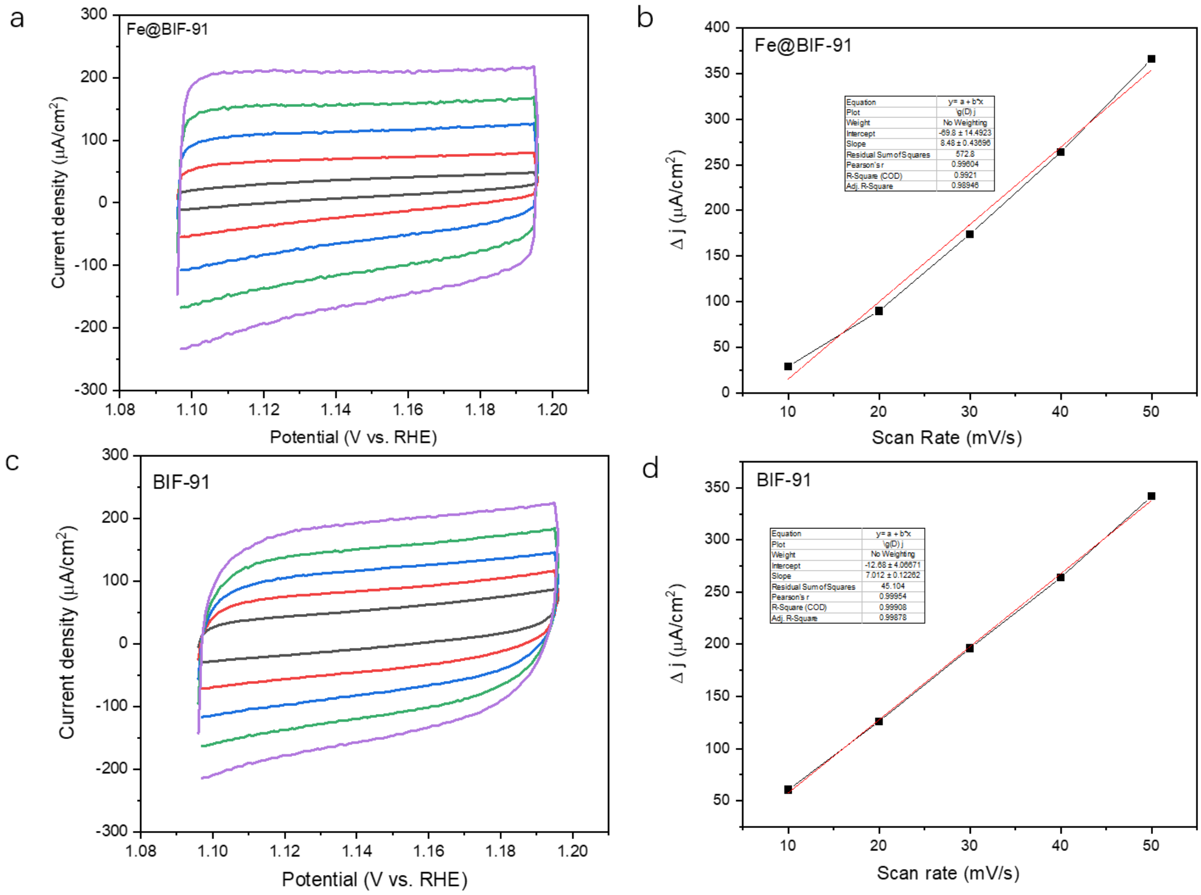

Figure S7. CV curves in the non-Faradaic zone with scan rates of 10, 20, 30, 40 and 50, mV s^{-1} and corresponding fit for **Fe@BIF-91** and **BIF-91** electrocatalysts.

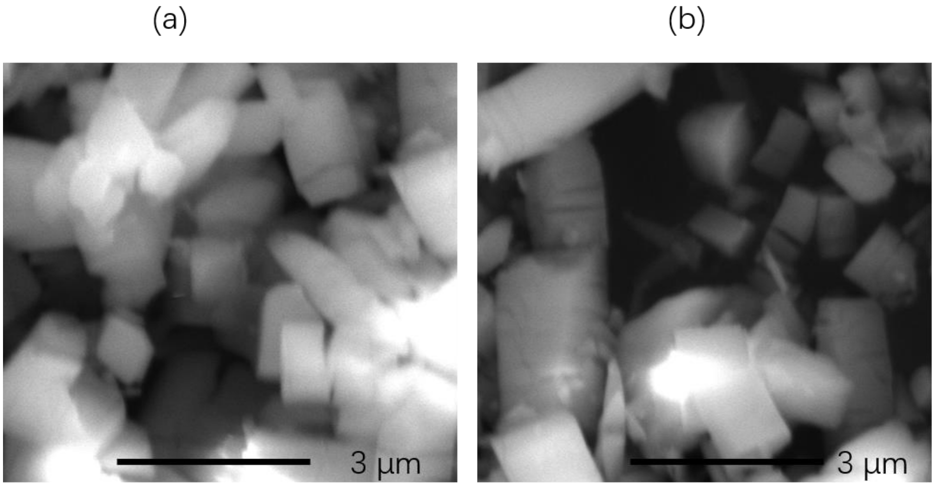

Figure S8. SEM morphology of the Fe@BIF-91 catalysts (a) before and after (b) the electrocatalytic tests.

Table S1. Summary of crystalographic data of **BIF-91**.

**BIF-91**	
Chemical formula	C_{18} H_{14} B Co N_8 O_2
Formula mass	444.11
Space group	P 21/c
a/Å	8.6140(5)
b/Å	14.5437(8)
c/Å	16.3657(8)
α/°	90.00
β/°	103.245(6)
γ/°	90.00
Volume/$Å^3$	1995.75(19)
Z, calculated density(g cm^{-3})	4, 1.478

F(000)	904.0
R_{int}	0.0398
GOF on F_2	1.02
R_1	0.0887 [a]
wR_2	0.263
CCDC Number	1870892

[a] $R_1 = \Sigma(|Fo| - |Fc|)/\Sigma|Fo|$, $wR_2 = [\Sigma w(Fo^2 - Fc^2)^2/\Sigma w(Fo^2)^2]^{0.5}$

Table S2. OER activity of **Fe@BIF-91** compared with reported MOF-based electrocatalysts.

**Catalyst**	**Onset potential**	**Overpotential at 10 mA cm^{-2}**	**Substrate**	**Ref**
Fe_3-Co_2<br>pH = 13	1.43	237	Cu Foil	1
Co-ZIF-9<br>pH = 13	NA	510<br>(1 mA cm^{-2})	FTO glass	2
Co-WOC-1<br>pH=13	1.62	390<br>(1 mA cm^{-2})	GC	3
FeTPyP-Co<br>pH = 13	1.47	351<br>(1 mA cm^{-2})	Au	4
USTA-16<br>pH = 14	1.60	408	GC	5
Co/MIL-100(Fe)<br>pH = 13	1.58	734<br>(5 mA cm^{-2})	GC-RDE	6
Co/MIL-101(Cr)<br>pH = 13	1.53	477	GC-RDE	7
UiO-67-[RuOH2]<br>pH = 6.2	1.96	818<br>(0.15 mA cm^{-2})	FTO	8

Fe@BIF-89<br>pH = 14	1.49	310	Ni-foam	9
Fe@BIF-91<br>pH = 14	1.50	351	GC-RDE	This work

References

[1] J. Q. Shen, P. Q. Liao, D. D. Zhou, C. T. He, J. X. Wu, W. X. Zhang, J. P. Zhang, X. M. Chen, J. Am. Chem. Soc. **2017**, 139, 1778.

[2] S. Wang, Y. Hou, S. Lin, X. Wang, Nanoscale **2014**, 6, 9930.

[3] P. Manna, J. Debgupta, S. Bose, S. K. Das, Angew. Chem. Int. Ed. **2016**, 55, 2425.

[4] B. Wurster, D. Grumelli, D. Hotger, R. Gutzler, K. Kern, J. Am. Chem. Soc. **2016**, 138, 3623.

[5] J. Jiang, L. Huang, X. Liu, L. Ai, ACS Appl. Mater. Inter. **2017**, 9, 7193.

[6] H. Wang, F. Yin, G. Li, B. Chen, Z. Wang, Int. J. Hydrogen Energy, **2014**, 39, 16179.

[7] X. B. He, F. X. Yin, G. R. Li, Int. J. Hydrogen Energy **2015**, 40, 9713.

[8] B. A. Johnson, A. Bhunia, O. Sascha, Dalton Trans. **2017**, 46, 1382.

[9] T. Wen, Y. Zheng, C. C. Xu, J. Zhang, M. Jaroniec, S. Z. Qiao, Mater. Horiz. **2018**, 5, 1151.
